# Supplementary material for: Unexplored photoluminescence from bulk and mechanically exfoliated few layers of Bi2Te3
Source: Sci Rep. 2018 Jun 15;8:9205. doi: 10.1038/s41598-018-27549-0 (PMC6004008; doi:10.1038/s41598-018-27549-0)
Supplement: Supplementary file 1 — Supplementary Information [file 41598_2018_27549_MOESM1_ESM.doc]

**Supplementary Information**

**Unexplored photoluminescence from bulk and mechanically exfoliated few layers of Bi_2_Te_3_**

Bipin Kumar Gupta*^,1^, Rabia Sultana^1, 2^, Satbir Singh^1, 2^, Vijeta Singh^1,2^ , Geet Awana^1,3^, Anurag Gupta^1^, Bahadur Singh^4^, A. K. Srivastava^1^, O.N. Srivastava^5^, S. Auluck^1^ and V.P.S. Awana*^,1^

*^1^CSIR- National Physical Laboratory, Dr K S Krishnan Road, New Delhi, 110012, India*

*^2^Academy of Scientific and Innovative Research (AcSIR), CSIR-National Physical Laboratory Campus ,Dr K S Krishnan Road, New Delhi 110012, India*

*^3^Department of Physics and Astrophysics Delhi University, New Delhi-110007, India*

*^4^Department of Physics, Indian Institute of Technology, Kanpur, U.P.-208016, India*

*^5^Department of Physics, Banaras Hindu University, Varanasi, U.P. - 221005, India*

***Correspondence to** [**bipinbhu@yahoo.com**](mailto:bipinbhu@yahoo.com) **(B.K.G.) and** [**awana@nplindia.org**](mailto:awana@nplindia.org)**(V.P.S.A.)**

***
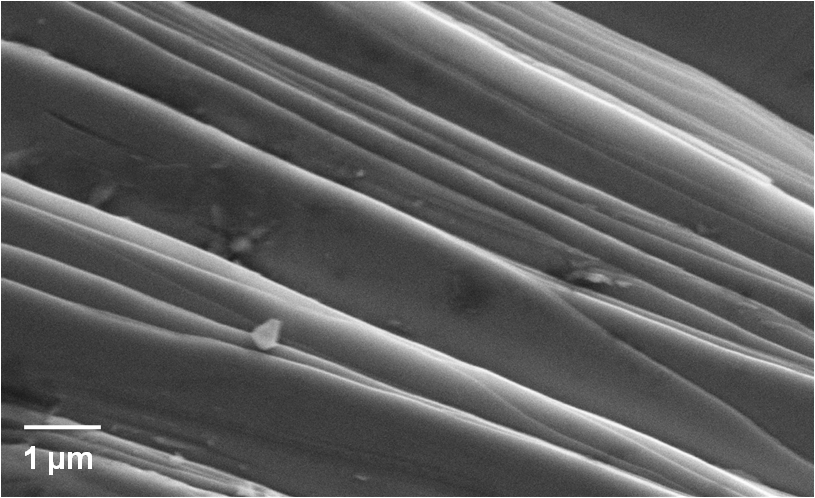
***

**Figure S1**. SEM micrograph of Bi_2_Te_3_ clearly shows the layered structure.

**
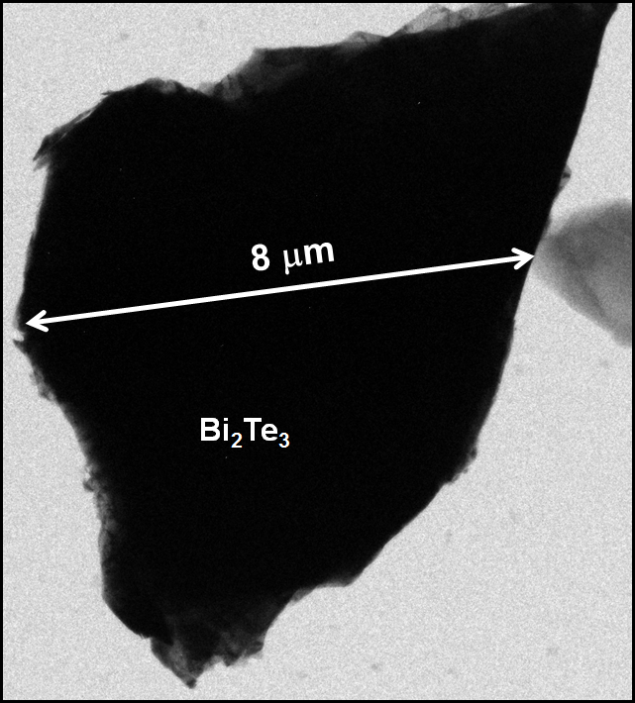
**

**Figure S2.** TEM micrograph of bulk Bi_2_Te_3_.

**
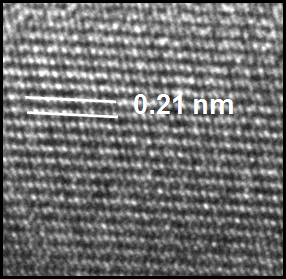
**

**Figure S3.** HRTEM image of bulk Bi_2_Te_3_ shows well resolved lattice fringes. The inter-planar spacing is 0.21 nm.

**
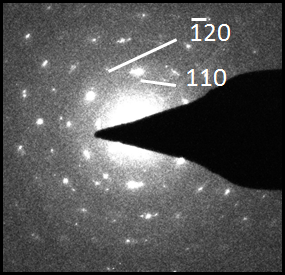
**

**Figure S4.** SAED pattern of bulk Bi_2_Te_3_ with well indexed diffraction spots.


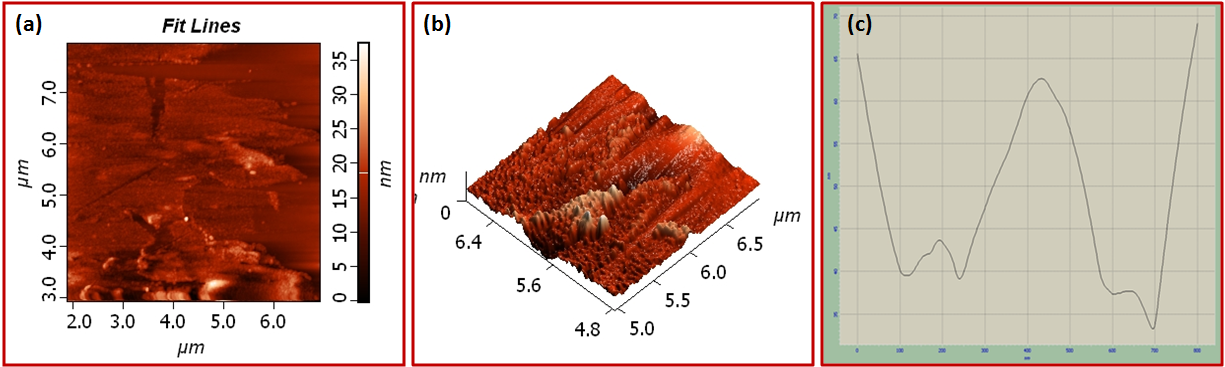


**Figure S5.** AFM micrograph of the mechanically exfoliated few layers of Bi_2_Te_3_: (a) represents the phase micrograph of few layers of Bi_2_Te_3_, where the line-scan profile is a 2D view, (b) exhibits 3D view of few layers of Bi_2_Te_3_ and (c) represents the line-scan profile, which clearly shows a thickness around (18±2) nm with wrinkle surface.


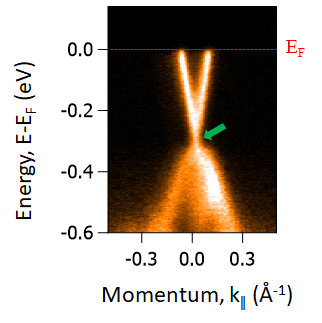


**Figure S6.** Energy distribution map (EDM) measured using ARPES depicting a Dirac point located at a binding energy of 300 meV below the Fermi level as shown by the green arrow on the EDM.

**Figure S7.** Reflectance spectrum of mechanically exfoliated few layers of Bi_2_Te_3_.
